# Location, Isolation, and Identification of Mesenchymal Stem Cells from Adult Human Sweat Glands

**DOI:** 10.1155/2018/2090276

**Published:** 2018-06-10

**Authors:** Yingzhi Ma, Meirong Li, Jinyu Liu, Chuanchao Pang, Jianqing Zhang, Yulin Li, Xiaobing Fu

**Affiliations:** ^1^Wound Healing and Cell Biology Laboratory, Institute of Basic Medical Science, Chinese PLA General Hospital, 28 Fu Xing Road, Beijing 100853, China; ^2^Department of Burn and Plastic Surgery, China-Japan Union Hospital, Jilin University, 26 Xiantai Street Changchun 130033, China; ^3^Department of Toxicology, School of Public Health, Jilin University, 1163 Xinmin Street, Changchun 130021, China; ^4^Department of Dermatology, the Second Hospital, Jilin University, 218 Zhiqiang Street, Changchun 130041, China; ^5^Department of Emergency Surgery, the Fourth Hospital, Jilin University, 91 Dongfeng Street, Changchun 130011, China; ^6^Key Laboratory of Pathobiology, Ministry of Education, Norman Bethune College of Medicine, Jilin University, 126 Xinmin Street, Changchun 130021, China

## Abstract

Sweat glands (SGs) are spread over almost the entire surface of the human body and are essential for thermoregulation. Theoretically, tissue-specific stem cells (TSSCs) are excellent candidate cells for the regeneration of SGs due to their genetic stability and differentiation ability. Herein, we attempted to isolate TSSCs derived from adult human sweat glands (ahSGs). ahSGs were localized and identified by H&E staining, double immunofluorescence staining, transmission electron microscope (TEM), and immuno-TEM. We found a population of cells with stem cell characteristics (SGSCs), located in basal myoepithelial cells of the secretory portion of the solenoid bulb. The SGSCs expressed alpha-smooth muscle actin (*α*-SMA) and showed the typical characteristics of mesenchymal stem cells (MSCs), with a positive antigen profile for CD44, CD73, CD90, and CD105, and had the multilineage differentiation potential to osteoblasts and adipocytes. In addition, the isolated *α*-SMA positive cells remained stably phenotypic and proliferative cycles at passage 12. This is the first report of successful isolation of MSC-like cells from ahSGs, which may contribute to wound repair and SG regeneration.

## 1. Introduction

In humans, SGs (eccrine glands) are by far the most abundant glandular structures in the body. They are essential for thermoregulation [1, 2]. When SGs cannot regenerate after a severe skin injury, such as serious burns and other reasons, failure to regulate body temperature in hot climates or during exercise can lead to hyperthermia, stroke, and even death. Therefore, it is necessary to induce SG regeneration after injuries.

MSCs are a potential therapeutic strategy for many diseases, such as diabetes, heart disease, and wound healing [3–6]. They can be isolated from a variety of human sources, including the bone marrow, fat tissue, umbilical cord, muscle [7], and hair follicles. Our group has been studying SG regeneration using stem cells for years. We previously showed that human bone marrow MSCs could be induced to resemble an SGC phenotype and that SGs could be regenerated in wounds after transplantation of these SGC-like cells [8]. Transplantation of MSC-induced SGCs could solve the problem of SG depletion after deep burns in the future. MSCs from other tissues may represent a stem cell reservoir for future use in the regeneration of SGs [9–11].

However, many experiments suggest variations in the differentiation capacity of MSCs from different tissue sources [12]. There have been studies on human hair follicle MSCs, which act in the metabolism of hair and repair of skin damage and are applied to cell therapies and regenerative medicine [13, 14]. SGs and hair follicles are appendages of the skin, and their embryonic development is similar. Therefore, we speculated that there might be MSCs in SGs, which could differentiate more efficiently into SGs and promote SG regeneration. However, research concerning isolation of SCs from SGs is infrequent. Current results have shown SCs, including SGSCs remaining in the quiescent state to maintain their undifferentiated state so they can provide homeostasis and function to repair tissue damage if necessary [15–17]. Most *in vivo* studies have focused on the contribution of SGCs to epidermal repair after superficial skin injuries [18]. Although these studies suggest that SGs contain SCs, precise localization and characterization of TSSCs from ahSGs has not been addressed so far.

Here, we investigate the location, isolation, culture, and identification of SCs derived from ahSGs. The aim of this work is to explore the existence of SGSCs and establish a technology platform for separation and cultivation of TSSCs from ahSGs, which will provide a new source for MSCs and prompt new ways for promoting wound repair and SG regeneration.

## 2. Materials and Methods

### 2.1. Skin Samples

Samples were obtained from full thickness skin of the knee, abdomen, palm, and forehead from adult humans (age range: 19–51 years old) during plastic and cosmetic or surgical operation after obtaining permission from the ethics commission of the Jilin University and informed consent by patients. Detailed information about the patient is listed in Table S1.

### 2.2. Antibodies

For immunofluorescence, immunoelectron microscopy and FCM (flow cytometry; BD Bioscience, USA), the antibodies are listed in Table S2.

As a secondary antibody, we used FITC-conjugated polyclonal goat Fab fragments directed to mouse and RITC-conjugated polyclonal goat Fab fragments directed to rabbit immunoglobulins (1 : 100; Bioss, China).

### 2.3. Histological and Immunofluorescence Staining Analysis

After dewaxing and hydration, sectioned samples were blocked with 10% bull serum albumin (BSA; Sigma, USA) for 30 minutes. Sections were incubated with primary antibodies, Carcinoembryonic antigen (CEA)/*α*-SMA and cytokeratin 15 (CK15)/*α*-SMA at 4°C overnight. Sections were then incubated with FITC/RITC-conjugated secondary antibodies for 1 h at room temperature. Nuclei were stained with Hoechst 33342 (Invitrogen, USA). Fluorescence signals were detected by laser scanning confocal microscopy (LSCM; Olympus, Japan).

### 2.4. TEM

For TEM analysis, tissue blocks were prefixed in 0.01 M phosphate-buffered saline (PBS) (pH 7.3) containing 2.5% glutaraldehyde for 2 hours, washed in PBS, postfixed with an aqueous solution of 1% OsO_4_ and 1.5% K_4_Fe(CN)_6_ for 1 hour, dehydrated, and finally embedded in EPON 812 (Catalys, Switzerland). Ultrathin sections (approximately 50–70 nm) collected on copper grids were contrasted with 4% uranyl acetate and 3% lead citrate and examined with TEM (TEI, USA). Immunoelectron microscopy was used to detect the expression of *α*-SMA in ahSG secretory portion by 10 nm colloidal gold (Bioss, China) as a secondary antibody.

### 2.5. Isolation of ahSG Solenoid Bulbs

Skin samples were stored in 0.01 M PBS containing 100 *μ*g/ml penicillin and 100 *μ*g/ml streptomycin (HyClone, USA) at 4°C within 24 h prior to use. A portion of the skin sample was fixed in 10% formalin for the preparation of paraffin sections. ahSG solenoid bulbs were located in the dermal reticular layer, closely associated with the subcutaneous tissue, surrounded by loose connective tissue and fat. By surgical dissection, the subcutaneous tissue wrapped in the outside of ahSG solenoid bulbs was removed under aseptic conditions. Skin tissue containing ahSG solenoid bulbs were cut into approximately 1 mm^3^ small pieces, digested in DMEM/F-12 (1 : 1) medium (Gibco, USA) containing 2% (g/ml) collagenase II (Invitrogen, USA) at 4°C. After 6 to 8 hours, ahSG solenoid bulbs were extracted from the skin tissue under a stereomicroscope.

### 2.6. Isolation and Purified Culture of ahSGCs

Extracted fragments were washed and cultured in DMEM/F-12 (1 : 1) medium (Gibco, USA) containing 10% (*v*/*v*) fetal bovine serum (FBS; HyClone, USA). After visible outgrowth, cells were removed by digestion with 0.1% trypsin and 1 mM EDTA (Invitrogen, USA) for 1 minute. When most of the fibroblast-like cells retracted and the SG epithelial cells remained adherent, an equal volume of culture medium containing 10% (*v*/*v*) FBS was added then continued to culture. The medium was replaced every 2-3 days. Cells were extensively propagated when they reached approximately 70–80% confluence. Cell morphology of different passages was observed under an inverted microscope (Olympus, Japan).

### 2.7. Immunofluorescent Cytochemical Staining Analysis

Cells on coverslips were fixed in 4% paraformaldehyde for 10 min permeabilized with 0.1% Triton X-100 (Sigma, USA) for 20 min at room temperature. Subsequently, 10% BSA was used to block nonspecific binding. Cells were then incubated with primary antibodies at 4°C overnight. Antibodies used were the following: *α*-SMA, CEA, CK15, FITC-CD44, FITC-CD73, FITC-CD90, FITC-CD105, FITC-CD34, and FITC-CD45. Samples of *α*-SMA, CEA, and CK 15 as primary antibodies were then incubated with secondary antibodies for 1 h at room temperature. Nuclei were stained with Hoechst 33342.

### 2.8. Immunophenotypic Characterization by FCM

Cells were harvested and resuspended in 0.01 M PBS at a density of 1 × 10^6^ cells/ml. Secondly, cells were fixed in 2% paraformaldehyde for 10 minutes. Samples of CEA, CK 15, and *α*-SMA as primary antibodies were permeabilized with 0.1% Triton X-100. All samples then used 10% (g/ml) BSA to block nonspecific binding for 20 min and were incubated with antibodies for 1 h at room temperature. Samples of CEA, CK15, and *α*-SMA as primary antibodies were in FITC-conjugated secondary antibody for 30 minutes at 4°C from light. After two washing step, cells were resuspended using 300 *μ*l PBS for FCM.

### 2.9. Cell Cycle Analysis

Cell cycle was analyzed by an annexin V-FITC/PI apoptosis detection kit (KeyGen, Nanjing, China). Cells were trypsinized into single cell suspensions and fixed with 70% ethanol at 4°C overnight. After being washed with PBS, cells were resuspended in staining solution containing RNase and propidium iodide and incubated for 30 min. DNA content was analyzed by FACS Calibur using Cell Quest software (BD Bioscience, USA).

### 2.10. Cell Proliferation Assays

Cell proliferation was measured using real-time cellular analysis (RTCA) iCELLigence analyzer (ACEA; Hangzhou, China) and EdU assay kit (RiboBio; Guangzhou, China). For RTCA, cells from different passages were digested and harvested with 0.1% trypsin and 1 mM EDTA. These cells were seeded onto E-Plate L8 culture plates with 2 × 10^4^cells per well and cultured for one week. According to the resistive variable data observed, cell proliferative capacity was analyzed by RTCA and cell growth curves were plotted from different passages (P). All experiments were completed in double, and two independent repeating experiments were performed.

For EdU incorporation assay, cells were cultured in 24-well plates for 48 h at 37°C, and then 50 *μ*M of EdU was added to each well. After culturing for additional 12 h at 37°C, cells were fixed with 4% paraformaldehyde for 15 min and then treated with 0.5% Triton X-100 for 20 min at room temperature for permeabilization. After washing with PBS three times, Apollo reaction cocktail was added to each well and cells were incubated for 30 min at room temperature. Then, cells were stained with Hoechst 33342 for 30 min and visualized under a fluorescent microscope (Olympus, Tokyo, Japan). EdU incorporation rate was expressed as the ratio of EdU positive cells (green) to total Hoechst 33342 positive cells (blue). Each sample was randomly counted three fields, and the average was calculated by the number of samples taken.

### 2.11. Induction of *α*-SMA Positive Cells from ahSGs to Differentiate into an Osteogenic and Adipogenic Lineage

Differentiation potential of ahSG *α*-SMA positive cells is evaluated by culturing cells in osteogenic and adipogenic media. Cells were induced for 4 weeks in osteogenic medium containing L-DMEM, 10% FBS, 0.1 *μ*M dexamethasone, 200 *μ*M ascorbic acid, and 10 mM b-glycerol phosphate [19]. After induction, osteoblasts were confirmed by cytochemical staining with 40 mM Alizarin red S dye (pH 4.2) to detect mineralized matrix according to the protocol described previously [20, 21]. Once cells reached 80% confluence, they were cultured in adipogenic medium for 2 weeks. The medium consisted of L-DMEM supplemented with 10% FBS, 1 *μ*mol/L dexamethasone, 50 *μ*mol/L indomethacin, 0.5 mM 3-isobutyl-1-methylxanthine, and 10 *μ*M insulin. At the end of the culture, the cells were fixed in 4% paraformaldehyde for 20 min and stained with oil red O solution to show lipid droplets in induced cells [19, 22]. All experiments were completed in triplicate, and three independent repeating experiments were performed.

### 2.12. Statistical Analysis

Analysis of variance was used to analyze the difference in immunophenotype among different samples and passages of cells. Data were expressed as mean ± standard deviation (SD). Results were considered significant at *P* < 0.05.

## 3. Results

### 3.1. Organizational Structural Characteristics of ahSG Solenoid Bulbs

**Figure 1:**
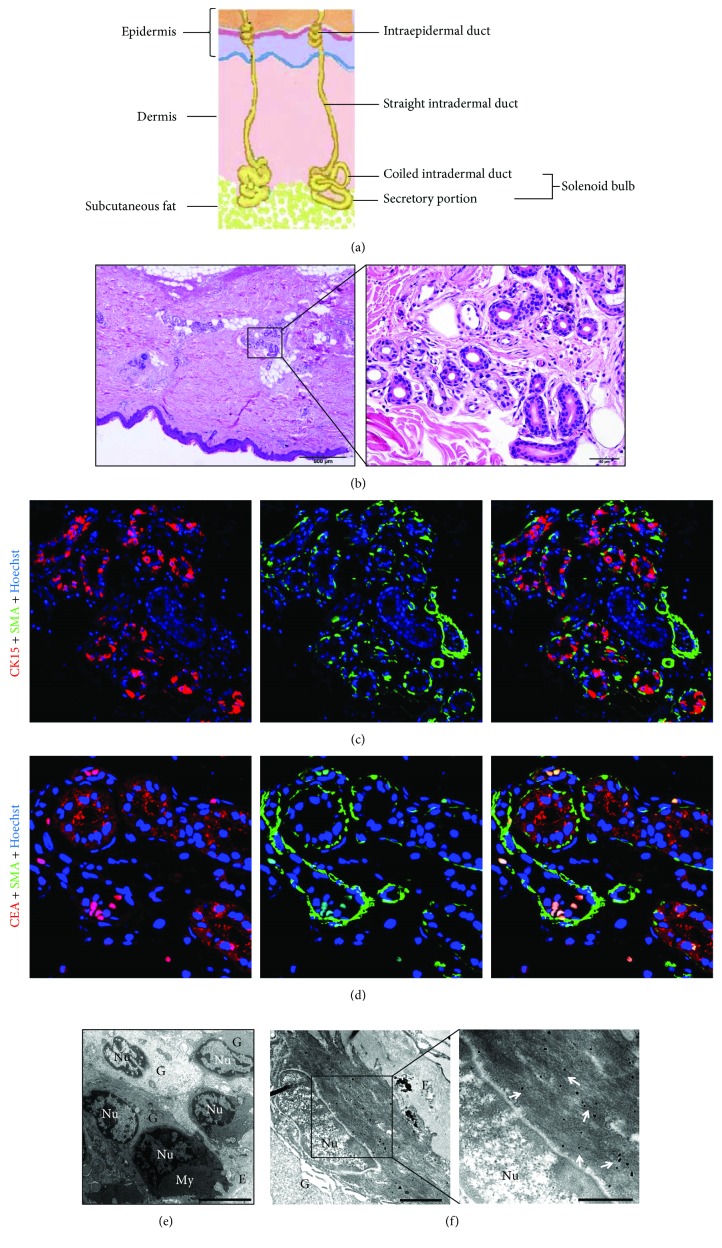
Histomorphology, immunocytochemical analysis, and ultrastructure of ahSGs *in vivo*. (a) Diagram showing each portion of ahSGs, which includes the intraepidermal, intradermal, and intraglandular duct and secretory portion. (b) H&E staining of the adult human skin (full thickness). The boxed area was magnified to determine that the solenoid bulb consisted of a duct and secretory portion. (c, d) Double immunofluorescence using the antibody combinations CEA or CK15 and *α*-SMA. The ahSG duct portion was positive for CEA and negative for CK15 (red) and *α*-SMA (green), whereas the secretory portion expressed CK15 (red) and *α*-SMA (green) with cell nuclei stained by Hoechst 33342 (blue). (e) TEM of the ahSG secretory portion. Myoepithelial cells were found in the outer portion of the glandular epithelial cells, and the pyramidal epithelial cells and flattened myoepithelial cells were closely related to each other. The outermost layer of the ahSG secretory portion was abundant ECM. Nuclei of myoepithelial cells were rich in heterochromatin that was darker, on the edge of the nucleus and around the nucleolus. Nu: nucleus; My: myoepithelial cell; G: glandular epithelial cell; E: ECM. (f) Immunoelectron microscopy of the ahSG secretary portion. The boxed area is magnified to visualize the myoepithelial cells. In the cytoplasm of the myoepithelial cells, the dispersive black dots are colloidal gold particles connected to *α*-SMA (white arrows indicate the colloidal gold particles). (b). Bar: 500 *μ*m and 50 *μ*m. (c) 400x. (d) 600x. (e) Bar: 5 *μ*m. (f) Bar: 2 *μ*m.

ahSGs are composed of four segments: intraepidermal duct, straight intradermal duct, coiled intradermal duct, and secretory portion (Figure 1(a)). The ahSG solenoid bulb includes the coiled intradermal duct and secretory portion. Via H&E staining, the solenoid bulb was found to be located in the connecting portion of the dermal and subcutaneous connective tissue (Figure 1(b)). The coiled intradermal duct consisted of a double layer of small cuboidal cells. The secretory portion appeared as irregularly arranged cells. An inner layer of epithelial cells in the ahSG secretory portion was surrounded by a layer of flattened myoepithelial cells.

To determine the cell composition and structural characteristics between the ahSG solenoid bulb and its surrounding tissue, we used double immunofluorescence with CK15 or CEA to detect skin histologic sections with *α*-SMA. *α*-SMA plays a central role in the binding of muscular contraction and is an important constituent of the myoepithelial cytoplasm. CK15 is considered to be a marker of very “young” proliferative human skin and is expressed in the basal layer and SCs of skin. CK15 is a useful indicator of epidermal homeostasis because it is almost exclusively expressed in basal keratinocytes in the homeostatic epidermis [23]. CEA, which is widely expressed in archenteric cancers and the digestive tract of a normal embryo, is expressed in ahSGs [24]. The results showed expression of CEA in SG epithelial cells, selective expression of CK15 in some of the epithelial cells, and expression of *α*-SMA in myoepithelial cells in the SG secretory portion (Figures 1(c) and 1(d)). *α*-SMA was expressed in almost all of the myoepithelial cells in the ahSG secretory portions and the vascular wall around them. Note that the coiled intradermal duct region expressed only CEA without *α*-SMA and CK15 expression.

Using TEM, we studied the features of the ahSG secretory portion at the ultrastructural level (Figure 1(e)). SG epithelial cells rested on myoepithelial cells or directly on the basal lamina. Myoepithelial cells contained large amounts of contractile actin filaments, which were arranged in parallel and aligned. Their nuclei were rich in heterochromatin. The ahSG secretory portions were connected by tight junctions (Figure S1). Next, we utilized immunoelectron microscopy to further ensure that the site of *α*-SMA was positive for SGSCs in the secretory portion (Figure 1(f)). On the basal side of the secretory portion, dispersive black colloidal gold particles were visible in the cytoplasm of myoepithelial cells in addition to the presence of abundant extracellular matrix (ECM).

*α*-SMA positive cells were found only in the ahSG secretory portion, tightly connected to the epithelial cells, and surrounded by rich ECM. This structure provided a basis for separating the SG solenoid bulbs from skin tissues.

### 3.2. Isolation and Primary Culture of ahSG Solenoid Bulbs

**Figure 2:**
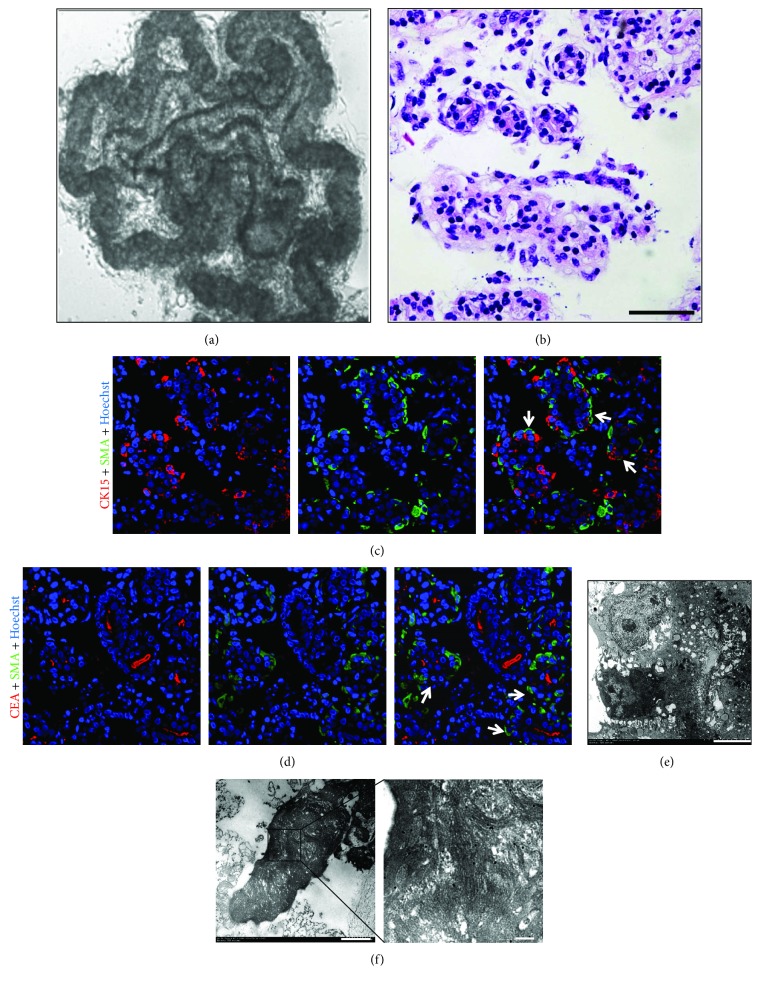
Histomorphology, immunocytochemical analysis, and ultrastructure of a detached solenoid bulb of ahSGs. (a) Phase contrast image of a detached ahSG solenoid bulb via phase contrast microscopy. (b) H&E staining of secretory and duct portions of the detached ahSG solenoid bulb. (c, d) Double immunofluorescence of the detached ahSG solenoid bulbs using antibodies against the following: CK15 ((c): red), CEA ((d): red), and *α*-SMA ((c, d): green) with cell nuclei stained by Hoechst 33342 (blue). White arrows indicated SMA positive cells. (e) The detached ahSG secretory portion by TEM. The ECM on the outside of the secretory portion of the detached ahSGs disappeared. (f) The detached ahSG secretory portion was observed by immunoelectron microscopy with anti-*α*-SMA as the primary antibody. Nu: nucleus; Myo: myoepithelial cells. White arrows indicated *α*-SMA positive immunogold labeling. (a) 40x. (b) Bar: 50 *μ*m. (c, d) 400x. (e) Bar: 5 *μ*m. (f) Bar: 2 *μ*m.

By H&E staining and TEM, we collected the subcutaneous tissue from full thickness skin, which was rich in solenoid bulbs which were cut into pieces, then were immersed in collagenase solution. The SG solenoid bulbs were selected under a stereomicroscope. These bulbs contained the thinner duct and the slightly swollen secretory portion that was associated with different refractivities (Figure 2(a)). H&E staining of separated SG solenoid bulbs showed that they were clearly dissociated from adjacent connective tissue (Figure 2(b)). Because of the enzymatic digestion, the integrity of the solenoid bulbs structure had been compromised. A small amount of connective tissue which located between the SG secretory portion and the duct, including the blood vessel, had disappeared. Immunofluorescence showed that CK15 and CEA were located in the lumen and *α*-SMA in the basement, consistent with marker expression of ahSGs *in vivo* (Figures 2(c) and 2(d)). No vascular tissue was found on H&E staining or by an immunofluorescence test. Based on TEM and the immunogold assay, the results were the same as those obtained *in vivo* (Figures 2(e) and 2(f)). Therefore, we ensured that the solenoid bulbs were integrally isolated from adult human skin, including *α*-SMA positive cells, CK15- or CEA-positive cells, and duct cells with negative expression of *α*-SMA, CK15, and CEA.

**Figure 3:**
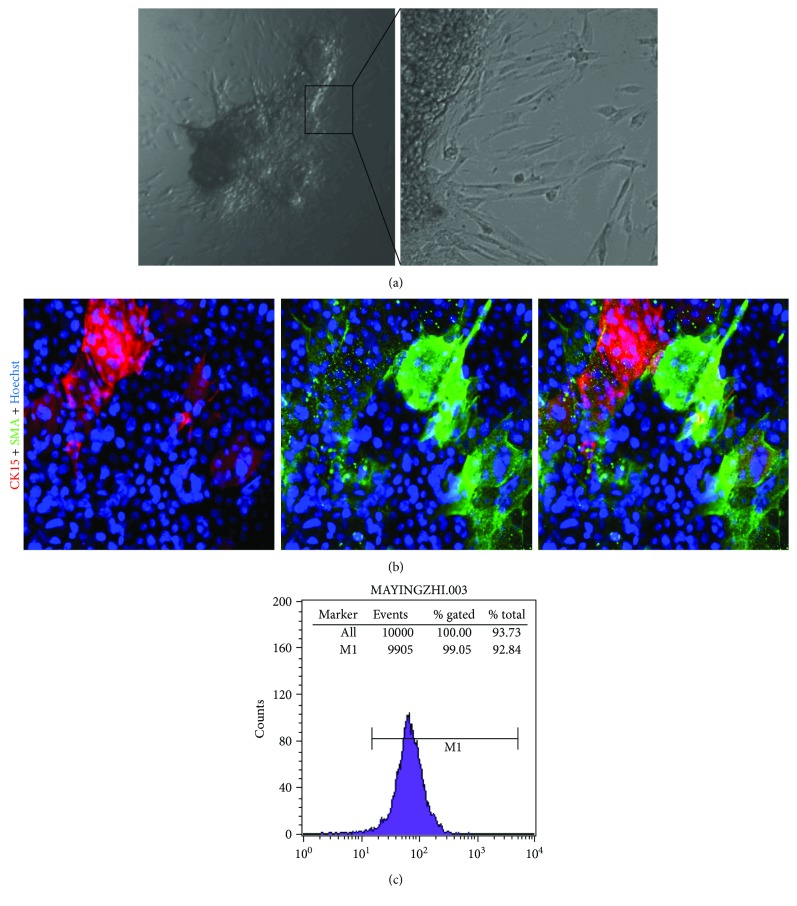
*In vitro* tissue culture from detached ahSG solenoid bulbs. (a) Typical morphology of different cells growing out from an ahSG fragment. The boxed area was magnified to visualize the fibroblast-like cells and epithelioid cells wrapped around them. (b) Double immunofluorescence of the primary cells growing out from the ahSG fragment using antibodies against CK15 and *α*-SMA. The cells were stained with antibodies to K15 (red) and *α*-SMA (green) with cell nuclei stained by Hoechst 33342 (blue). (c) FCM showing *α*-SMA expression by the hSG secretory cells. (a) 40x and the box is 100x. (b) 400x.

Following tissue culture of these SG fragments for 2–5 days, there were two types of cells that grew from the explants, including typical epithelial cells (assuming a cobblestone morphology) and long spindle fibroblast-like cells (Figure 3(a)). The immunofluorescence results showed that the fibroblast-like cells expressed *α*-SMA, and the part of epithelioid cells expressed CK15 (Figure 3(b)). There was no coexpression of *α*-SMA and CK15 in the same cell. By immunofluorescence staining with CK15 and *α*-SMA as primary antibodies, some epithelial cells were CK15 positive, and all fibroblast-like cells were *α*-SMA positive (Figure 3(b)). Interestingly, we found that different morphological cells have different sensitivity to trypsin. Compared with the epithelial cells, the *α*-SMA positive cells were more sensitive to trypsin and were easily digested. Therefore, we can use this differential digestion method to obtain purified *α*-SMA positive cells. It has been proved that after approximately 1 week in primary culture, the fibroblast-like cells were digested by trypsin and had high purity of expression of *α*-SMA by FCM *in vitro*. The result was that over 99% of the cells expressed *α*-SMA but none expressed CK15 by FCM.

Given the current results showing that only myoepithelial cells were *α*-SMA positive in the isolated SGs, the *α*-SMA positive cells could only be derived from ahSG myoepithelium cells. In the next experimental series, we explored the biological characteristics of these *α*-SMA positive cells from ahSGs.

### 3.3. Identification of Relevant Characteristics of Stem Cells in the *α*-SMA Positive Cells from ahSG Fragments

**Figure 4:**
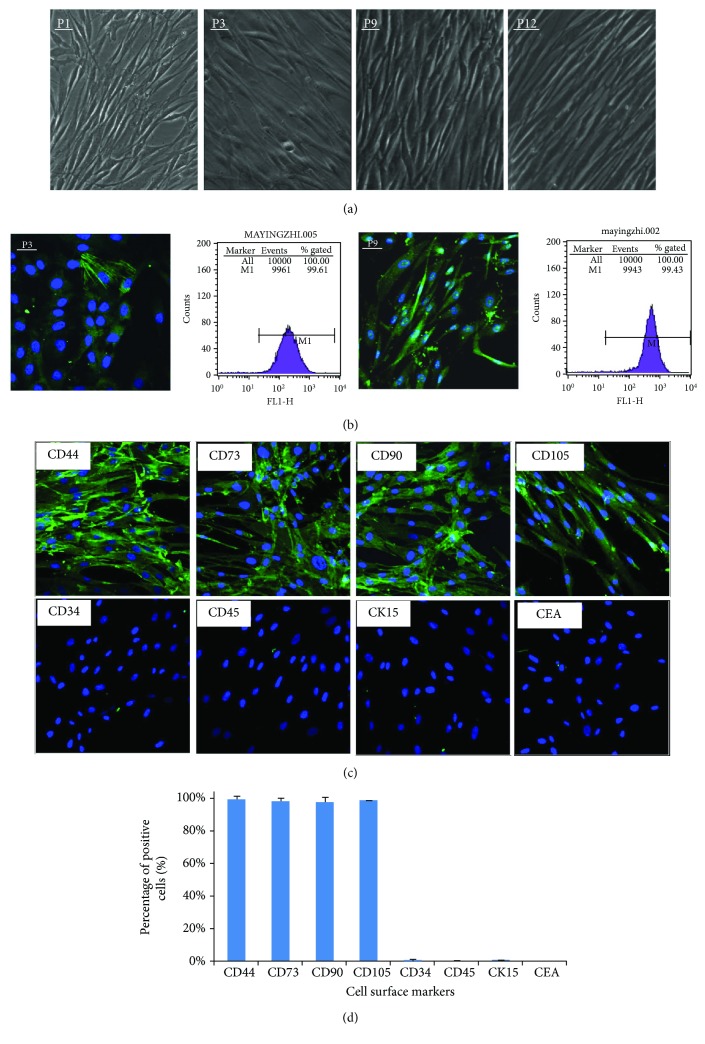
Morphological characteristics and phenotypes of *α*-SMA positive cells from ahSGs at different passages (400x). (a) Cells at different passages had the same long fusiform shape (i.e., fishes or whirlpool) when they grew to 80% to 90% as observed under an inverted microscope. (b) Expression of *α*-SMA in the P3 and P9 generations of the cells by immunofluorescence and FCM. (c) Immunophenotypic characterization of MSC-relative markers in the cells by immunofluorescence. (d) FCM showed that the cells were positive for CD29, CD44, CD73, and CD105 with all expression rates up to 95% but negative for CD34, CD45, CK15, and CEA.

Cells were passaged every 4-5 days for a maximum of 12 passages without displaying any morphological alterations under an inverted microscope (Figure 4(a)). Primary and passaged cells all displayed typical fibroblast-like morphological features with a fusiform shape, as a whirlpool or fish-like arrangement. Nearly all cells expressed *α*-SMA according to immunofluorescence, and 97.00% ± 3.70% was positive by FCM (Figure 4(b)). Therefore, high purity *α*-SMA positive cells from ahSG myoepithelial cells were obtained and stable for 12 generations. It has been reported that myoepithelial cells are the source of SGSCs [25].

Based on the cell morphology and tissue source, we identified SC-relevant morphologic properties of these cells. CD44, CD73, CD90, and CD105 are accepted as markers to identify MSCs. First, we used immunofluorescence to quantify the MSC-related immunophenotype of *α*-SMA positive cells from ahSGs. The cells expressed CD44, CD73, CD90, and CD105; however, they were negative for the hematopoietic lineage markers CD31, CD45, and CK15, and CEA expression in ahSG epithelial cells was not detected (Figure 4(c)). Second, we repeated the assay using FACS for different samples and passages of cells. The FACS results were consistent with the immunofluorescence results: more than 90% of cells expressed CD44 (98.78% ± 2.13%), CD73 (97.99% ± 1.91%), CD90 (97.34% ± 3.06%), and CD105 (98.36% ± 0.29%) but not CD34 (0.79% ± 0.36%), CD45 (0.47% ± 0.20%), CK15 (0.61% ± 0.13%), or CEA (0.36% ± 0.13%) (Figure 4(c) and Figure S2). Results were considered significant at *P* < 0.05. Therefore, *α*-SMA positive cells from ahSGs had the same immunophenotype as MSCs derived from other tissues, such as the bone marrow.

**Figure 5:**
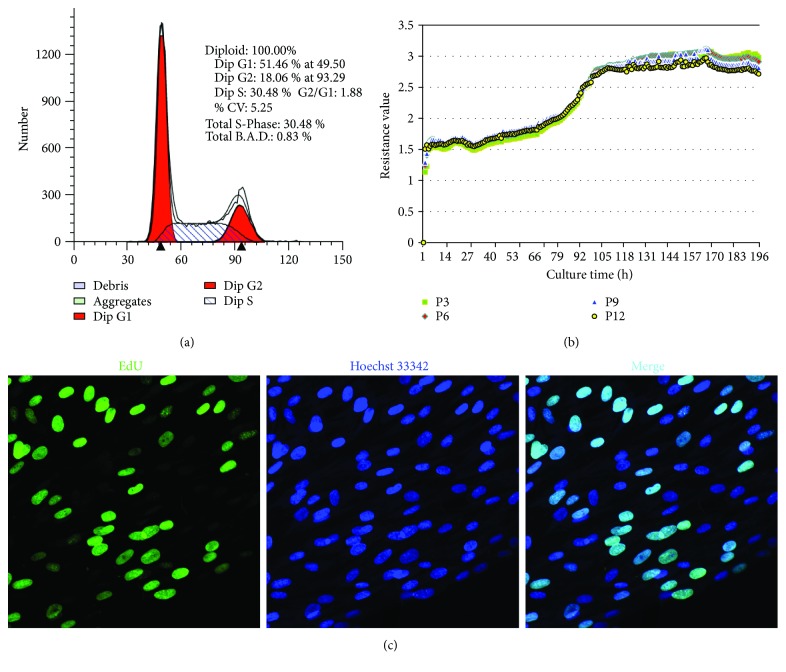
Reproductive activity of *α*-SMA positive cells from ahSGs. (a) Cell cycle of P6 tested by FACS. (b) Cell growth curve of P3, 6, 9, and 12 by RTCA. (c) Cell proliferation by EdU incorporation assay. EdU-labeled replicating cells are green, and all cell nuclei are blue under fluorescence microscopy (400x).

To detect cell proliferation and self-renewal ability, we collected cell cycle measurements. The DNA contents were detected by FACSCalibur and analyzed with Cell Quest software for P3 and P9 passaged cells (Figure 5(a)). The results showed that the ratio of cells in the DNA synthesis phase (S phase and G2/M phase) (the active proliferative phase) was 15.1 ± 2.9%, with the remaining cells in the G0/G1 phase (quiescent phase, 84.9% ± 2.9%) (Figure 5(a)). Next, the growth kinetics of the cells was determined by RTCA. All of the growth curves from four different passages (P3, P6, P9, and P12) displayed an initial quiescent phase during the first 2 days in culture, a log phase at an exponential rate from 3 to 5 days, followed by a plateau phase. There was no significant difference in growth rate among different passages of cells (Figure 5(b)). The cells all showed powerful and stable reproductive activity from P3 to P12. Next, we investigated the proliferative status of *α*-SMA positive cells with the relative number of cells in the S phase examined by EdU labeling. After the incorporation of EdU for 24 h, there were 60.24 ± 6.65% cells that positively expressed EdU by immunofluorescence and were undergoing division and proliferation during that period (Figure 5(c)). The EdU incorporation assay provided us with an intuitive view of the state of cell division and proliferation.

**Figure 6:**
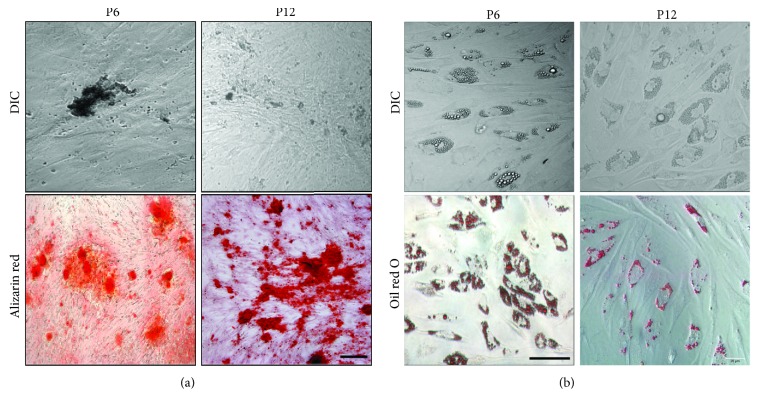
Differentiation potential of *α*-SMA positive cells from ahSGs. (a) Osteogenic differentiation was evaluated by culturing cells in osteogenic media and Alizarin red S staining. (b) Adipogenic differentiation was evaluated by culturing cells in adipogenic media and oil red O staining. (a) Bar: 100 *μ*m. (b) Bar: 50 *μ*m.

SCs have two requirements: self-renewal proliferation and differentiation potential. From the above results, we learned that the acquired *α*-SMA positive cells had a strong proliferation ability. To further characterize their multipotency, we tested the differentiation potency of these ahSG *α*-SMA positive cells for their osteogenic and adipogenic induction ability. When cultured in osteogenic medium for 2 weeks, *α*-SMA positive cells became polygonal and formed into a clustered arrangement; when cultured in osteogenic medium for 4 weeks, they appeared to differentiate into osteoblast-like cells. Alizarin red S staining was performed to detect the formation of bone nodules. It was shown that *α*-SMA positive cells had the ability to differentiate into the bone (Figure 6(a)). After adipogenic induction for 3 days, the cell morphology changed from a long spindle-shape into a polygonal shape. One week later, small bubble-shaped lipid droplets appeared in a portion of the cells. The size of the lipid droplets increased after two weeks, and most of the differentiated cells appeared red after oil red O staining of the lipid droplets throughout the cytoplasm (Figure 6(b)). This showed that the cells had the ability to differentiate into fat.

From these data, we concluded that *α*-SMA positive cells from ahSGs could proliferate well and self-renew, and they had the potential to differentiate into fat and bone, similar to the biological characteristics of MSCs.

## 4. Discussion

TSSCs have stable differentiation and proliferation ability and thus maintain tissue homeostasis [26, 27]. They play an incomparable role to other source cells in the treatment of diseases and tissue repair [28, 29]. SGs, as skin appendages, are a type of mini organ with important functions. It is difficult to regenerate after injury. In recent years, researchers have sought to discover an ideal stem cell source for cell therapy to repair and reconstruct SG in wounds. However, little progress has been made in SGSCs. However, it has been shown that there are SCs in SGs, and these SCs are attached to the basement membrane of the proximal acinar gland region of SGs and have the ability to promote damage repair and maintain homeostasis in a transgenic mouse model [25, 27, 30, 31].

Therefore, this study focused on exploring and separating SG-specific stem cells. We started with the tissue structure of SGs to determine possible sites of SCs located in SGs. By observing the structure of SGs, it was clear that there was an abundant heterochromatin in the nucleus of the myoepithelium, and these cells could be SCs. Meanwhile, SG solenoid bulbs were surrounded by rich ECM, and the inner cells were tightly connected by tight junctions (Figure S1). As the IF results show, the joint expression of *α*-SMA and CEA/CK15 was in the location of ahSG solenoid bulbs, but blood vessels only expressed *α*-SMA positive. This expression features provided a structural basis for the separation of ahSG solenoid bulbs from skin tissues. Then, we adopted enzyme digestion to efficiently separate SG solenoid bulbs to obtain SCs from ahSGs; this method could protect the activity of SGSCs and lead to subsequent purification of SCs. In previous studies, researchers have tried to isolate and culture of SGSCs; long spindle cells were occasionally present and were thought to be fibroblasts [32]. These cells were treated as miscellaneous cells and removed. We speculate that the specific anatomical location of SGSCs which mainly distributes around the SG and the enzyme digestion for a long time will affect the biological activity of these cells. This may be the main reason why SGSCs are difficult to obtain. In our experiment, we strictly controlled the digestion time of collagenase. Therefore, this method significantly reduced the damage to SGSCs, which led us to eventual successful separation of MSCs from ahSGs.

This tissue culture method had the advantages of being a simple method and could obtain a large number of cells; however, the obtained cell types were complex. Cells obtained by tissue culture of SG solenoid bulbs were of at least three types, namely, *α*-SMA positive myoepithelial cells and epithelial cells that were either CK15 positive or negative. The *α*-SMA positive cells were sensitive to trypsin and easy to separate from the epithelial cells. Isolated primary SGSCs were identified by *α*-SMA, and their positive rate was over 99%. This suggests that the *α*-SMA positive cells obtained from ahSGs had high purity. These cells were stable for more than 12 generations, during which the cell morphology, molecular phenotype, proliferative capacity, and differentiation potential all conformed to the biological characteristics of MSCs.

Our study is the first to successfully isolate MSCs from ahSGs with high purity; the MSCs were able to be amplified exponentially. It has been clearly determined that myoepithelial cells of the SG secretory portion are important sources of ahSG-MSCs. We completed isolation, culture, and identification of ahSGs from six skin specimens from different ages following the same experimental procedure and confirmed the existence of *α*-SMA positive multipotent stem cells in ahSGs. Here, we have demonstrated that *α*-SMA positive cells with MSC characteristics exist in SGs as a scattered population localized in the SG basal layer of the proximal acinar region. A technical platform for isolating MSCs from ahSGs was established.

Our findings additionally noted that some glandular epithelial cells from ahSG solenoid bulbs expressed CK15. CK15 is a marker of epidermal stem cells [33]; therefore, it is indicated that these CK15-positive cells could contain SG epithelial stem cells. However, further isolation and identification of cultured SG epithelial cells were not performed in this experiment because of the present isolation methods and limitations. MSCs from different sources have shown the ability to regenerate sweat glands after being induced. Therefore, we hypothesize that tissue-specific MSCs from SGs may also contribute to the SG regeneration. Therefore, its further differentiation ability, especially its ability to differentiate into SGs, promote skin regeneration, and promote the transformation of other cells into SGCs, will be demonstrated in our further study. Additionally, autologous cells are preferred in the transplantation setting, avoiding the risks of immune rejection. If SG myoepithelial cells and glandular epithelial cells can simultaneously be amplified and cultured from one individual, with the cells coming from the same individual, we may be able to build an unrejected SG. If that is the case, individual tissue reconstruction would be possible.

In conclusion, this study showed there were tissue-specific MSCs in ahSGs expressing *α*-SMA. Therefore, further studies are needed to investigate whether MSCs from ahSGs can directly promote SG regeneration or restore better SG function when interacting with SG epithelial cells. Our findings indicate that glandular epithelial stem cells could be present in adult sweat glands. TSSCs from ahSGs may provide new opportunities for SG regeneration. In the future, we will assume that TSSCs from ahSGs, including SMA positive MSCs, can be combined with SG epithelial cells to obtain artificial SGs similar to mature SGs by using 3D printing technology.

## Data Availability

The data used to support the findings of this study are available from the corresponding author upon request.

## Supplementary Materials

Supplementary Materials Figure S1: the inner cells of the ahSG secretary portion are tightly connected by tight junctions (white arrows) by TEM (Bar: 5 *μ*m). Figure S2: phenotypic characteristics of MSCs in the P6 and P12. Table S1: features of skin samples. Table S2: details of the antibodies used in this study.
