# Supplementary material for: Location, Isolation, and Identification of Mesenchymal Stem Cells from Adult Human Sweat Glands
Source: Stem Cells Int. 2018 Jun 10;2018:2090276. doi: 10.1155/2018/2090276 (PMC6015687; doi:10.1155/2018/2090276)
Supplement: Supplementary Materials — Figure S1: the inner cells of the ahSG secretary portion are tightly connected by tight junctions (white arrows) by TEM (Bar: 5 μm). Figure S2: phenotypic characteristics of MSCs in the P6 and P12. Table S1: features of skin samples. Table S2: details of the antibodies used in this study. [file 2090276.f1.doc]

**Supplementary Material**

**Figure S1** It shows that the inner cells of the ahSG secretary portion are tightly connected by tight junctions (white arrows) by TEM (Bar: 5 µm).


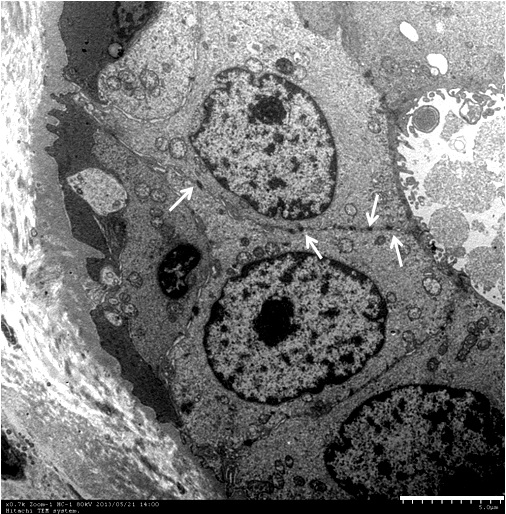


Figure S2 Figure S2 Phenotypic characteristics of MSCs in the P6 and P12 by FCM (A: P6; B: P12).


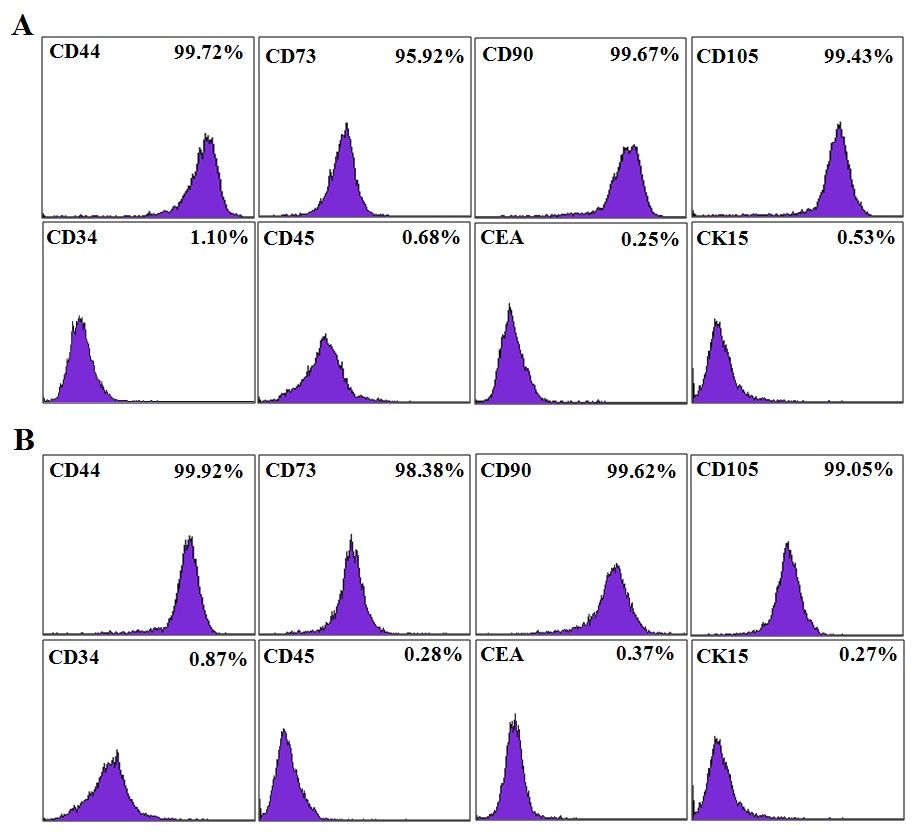


**Table S1 A table is about the features of skin samples.**

| Stamples | Time (y/m/d) | Source | Age | Sex | Biopsy site |
| --- | --- | --- | --- | --- | --- |
| 1 | 2012.04.01 | The Second Hospital, Jilin University | 58 Y | F | Forehead |
| 2 | 2012.05.04 | China-Japan Union Hospital l, Jilin University | 36 Y | M | Abdomen |
| 3 | 2012.07.16 | China-Japan Union Hospital, Jilin University | 21 Y | M | Hand |
| 4 | 2012.11.04 | China-Japan Union Hospital, Jilin University | 42 Y | M | Hand |
| 5 | 2014.06.26 | The Forth Hospital, Jilin University | 32 Y | M | Forearm |
| 6 | 2014.07.13 | The Forth Hospital, Jilin University | 19 Y | M | Knee |

(Abbreviation: y: years old; F: female; M: male)

**Table S2 Details of the antibodies used in this study.**

| Number | Name | Co. | Host species | Clone | Concentration | Dilution |
| --- | --- | --- | --- | --- | --- | --- |
| ab924 | Anti-Carcino Embryonic Antigen (CEA) | Abcam | Rabbit | Polyclonal | 0.20mg/ml | 1:100 |
| ab7817 | Anti-alpha smooth muscle Action (SMA) |  | Mouse | Monoclonal | 1 mg/ml | 1:100 |
| ab52816 | Anti-cytokeratin 15 (CK15) |  | Rabbit | Monoclonal | 0.029mg/ml | 1:50 |
| 555478 | FITC-anti-CD44 | BD | Mouse | Monoclonal | 20 μl/test | 1:100 |
| 555821 | FITC-anti-CD34 |  | Mouse | Monoclonal | 20 μl/test | 1:100 |
| 555482 | FITC-anti-CD45 |  | Mouse | Monoclonal | 20 μl/test | 1:100 |
| 555595 | FITC-anti-CD90 |  | Mouse | Monoclonal | 0.5 mg/ml | 1:100 |
| 561254 | FITC-anti-CD73 |  | Mouse | Monoclonal | 5 μl/test | 1:100 |
| 561443 | FITC-anti-CD105 |  | Mouse | Monoclonal | 5 μl/test | 1:100 |
